# Gut Bacterial Communities in Geographically Distant Populations of Farmed Sea Bream (*Sparus aurata*) and Sea Bass (*Dicentrarchus labrax*)

**DOI:** 10.3390/microorganisms6030092

**Published:** 2018-09-01

**Authors:** Eleni Nikouli, Alexandra Meziti, Efthimia Antonopoulou, Eleni Mente, Konstantinos A. Kormas

**Affiliations:** 1 Department of Ichthyology and Aquatic Environment, School of Agricultural Sciences, University of Thessaly, Volos 384 46, Greece; nikouli.eleni@gmail.com (E.N.); ameziti@gmail.com (A.M.); emente@uth.gr (E.M.); 2 Laboratory of Animal Physiology, Department of Zoology, School of Biology, Aristotle University of Thessaloniki, Thessaloniki 541 24, Greece; eantono@bio.auth.gr

**Keywords:** teleosts, intestine, bacteria, microbiota, aquaculture

## Abstract

This study investigated the profile of the autochthonous gut bacterial communities in adult individuals of *Sparus aurata* and *Dicentrarchus labrax* reared in sea cages in five distantly located aquaculture farms in Greece and determine the impact of geographic location on them in order to detect the core gut microbiota of these commercially important fish species. Data analyses resulted in no significant geographic impact in the gut microbial communities within the two host species, while strong similarities between them were also present. Our survey revealed the existence of a core gut microbiota within and between the two host species independent of diet and geographic location consisting of the *Delftia*, *Pseudomonas*, *Pelomonas*, *Propionibacterium*, and *Atopostipes* genera.

## 1. Introduction

Studies on fish gastrointestinal tract microbiota (GITM) are mostly focused on the isolation, identification and evaluation of microorganisms in farmed species. The main target of such studies is the possible use of these microorganisms as potential probiotics in order to promote fish growth and health [1]. With the advent of next generation sequencing technologies, results have demonstrated that fish GITM diversity shows higher complexity than originally considered [2]. Knowing the core microbiota (sensu [3]) is pivotal in predicting and further investigating the provided microbial services to the host [4], since these communities are important for the ecological understanding of the gut habitat and the functions of its microbes [5]. The investigation of co-occurrence patterns, including core and less frequent occurring microbes, has been shown to be extremely useful for depicting fundamental and keystone microbial species across same types of habitats-host in spatial and temporal scales [6]. Such approaches have shown that correlations between microbes and latitude can exist even for the human gut [7].

While dietary studies profiling the human gut microbiota pose certain limitations [8], sea cage farmed fish species can be a good model system to investigate fish core GITM since these populations are genetically homogeneous and consume a well-balanced diet that meets their nutritional requirements throughout their life cycle, while populations of the same species are reared in similar environmental conditions. For fish GITM, it has been suggested that these communities are not mere reflections of their host’s habitat but are rather shaped by host-specific selective forces [9]. In this study, we compared the GITM of *Sparus aurata* and *Dicentrarchus labrax* individuals originating from five distantly located aquaculture installations in Greece in order to reveal their core GITM, i.e., bacteria that occur across all samples regardless of location and supplied diet.

## 2. Materials and Methods

Adult individuals of *S. aurata* and *D. labrax*, weighing on average 451 ± 86.4 and 481.3 ± 165.5 (Table S1), respectively, were collected from five commercial aquaculture farms distantly located from each other in different areas in Greece (Figure S1). Fish were grown in sea cages and fed commercial diets (Table S2), and raised under similar husbandry conditions (temperature, pH, salinity, feeding frequency) throughout the rearing cycle. All samples were collected in September 2014 in order to limit possible seasonal variations. Fish were sacrificed by emersion on ice water, packaged in insulated boxes with melted ice (0 °C), and transferred to the laboratory within 6–24 h. Wet weight was measured and gut tissues were obtained by aseptic dissection and the intestinal content was squeezed out. The midgut from 4–6 individuals from each species (*n* = 2) originating from the same cage in every location (*n* = 5) was excised with sterile scissors and rinsed with sterile particle free seawater, as we targeted the resident gut microorganisms, i.e., epi and endobionts of the gut tissue cells, and not the ones associated with the ingested food. Gut samples were kept at −80 °C until further analysis. DNA was extracted directly from ca. 0.25 g gut tissue using the PowerMax Soil DNA Isolation kit (MoBio, Carlsbad, CA, USA) according to manufacturer’s protocol. The concentrations of extracted DNA (absorbance at 260nm) and purity (absorbance ratio 260/280) were measured using NanoDrop (ThermoScientific, Waltham, MA, USA). We analyzed the 16S rDNA gene diversity of gut bacteria from each individual sample, targeting the V3­V4 region by using 454 pyrosequencing with the primer pair S­D-Bact­0341­b­S­17 and S­D­Bact­0785­a­A­21 [10]. Samples were sequenced utilizing Roche 454 FLX titanium instruments and reagents after following manufacturer’s guidelines at the MRDNA Ltd. (Shallowater, TX, USA) sequencing facilities. Pyrosequencing reads were processed by the MOTHUR platform (Pat Schloss, University of Michigan, MI, USA; version 1.38) [11,12]. Only sequences with ≥250 bp and no ambiguous or no homopolymers ≥8 bp were considered for further analysis. All remaining sequences were binned in operational taxonomic units (OTUs) and were clustered using a 97% sequence similarity threshold. OTUs taxonomic classification was determined by the SILVA Incremental Aligner (SINA) online alignment service for small (16S) subunit ribosomal RNA [13], by setting minimum identity with query sequence 0.95 and by rejecting sequences below identity 80%. The sequences that could not be classified into any known phylum were assigned as “unclassified” from the SILVA database, release 130 [14].

Statistical analysis and graphical illustrations were performed using the PAlaeontological STudies (PAST) software [15] and the R Studio platform [16]. Macroecological patterns were calculated based on species area relationship (SAR) according to [17]. To evaluate host-specific dynamics, we applied the “DOC method” [18] by calculating the correlation between the overlap and dissimilarity of all OTUs for all the possible individual pairs from the five locations for each host species. To reveal microbial associations within the gut environment the network approach of [19] was used based on the ratio of positive to total correlations of the most dominant OTUs in individuals of *S. aurata* and *D. labrax*. Raw sequence data from this study have been submitted to the Sequence Read Archive (https://www.ncbi.nlm.nih.gov/sra/) with accession numbers SRR5161931 and SRR5803847, for *S. aurata* and *D. labrax*, respectively.

## 3. Results and Discussion

In this study, we analyzed the midgut bacterial diversity of farmed *Sparus aurata* and *Dicentrarchus labrax* individuals in order to determine members of the adult core microbiota of these commercially important fish species. Taking into account that microbiota are important in health and disease, revealing the core microbiota of a species would be important in order to explore how to achieve a beneficial collaboration between host and microbiota (for a review see [5]). The analyzed fish individuals had the following features: (a) common genetic origin, (b) very similar supplied commercial feed (Table S2), (c) origin from distant aquaculture farms (23–554 km between them), (d) similar age, and (e) were sampled within days. These criteria allowed us to assess the core microbiota of these animals by minimizing the effects of host genetics, nutritional state and environmental stressors (e.g., salinity, temperature) variability. In this study, core OTUs refer to the ones found in each individual midgut sample. The effect of the surrounding water was not studied since it is expected to be insignificant for the GITM diversity as shown previously [20,21,22,23]. A single water sampling on the same day of the midgut sampling of the investigated fish individuals, would not be so informative due to the following two factors: 1) marine bacterioplankton is characterized by strong variation in short (e.g., [24,25]) and longer [26,27,28,29] time scales, and 2) the life cycle of farmed *S. aurata* and *D. labrax* spans over several months. To the best of our knowledge, this is the first study combining all the above features for the gut bacterial communities of *S*. *aurata* and *D*. *labrax*. Floris et al. [30] investigated the gut microbiota of *S*. *aurata* at two coastal lagoons in Sardinia, Italy, but their study was based on older techniques with limited power to uncover the full extent of biodiversity. For *D*. *labrax*, there are a few relevant studies but were mainly focused on candidate probiotic’s evaluation [31,32,33,34] and the effect of alternative feed ingredients in gut microbiome [35].

Despite the low reads numbers in some samples (Table 1) rarefaction curves have reached a plateau (Figure S2), indicating satisfactory coverage of the existing bacterial OTUs. The effect of different aquaculture location on bacterial species richness was not important since OTUs richness between locations did not vary significantly (Figure S3). Each species had a rather defined bacterial community, with 10–21 OTUs accounting for ≥80% of the relative abundance per sample (Table 1).

All detected OTUs belonged to 11 different phyla (Figure S4), commonly occurring in fish gut [36,37] with *Proteobacteria*, *Firmicutes*, *Actinobacteria* dominating (>78%) across all samples. *Bacteroidetes*-related OTUs also occurred in all locations for both species but with lower contributions (Figure S4). The rest of the phyla (*Chloroflexi*, *Spirochaetae*, *Deinoccocus*-*Thermus*, *Cyanobacteria*, *Saccharibacteria*, *Gemmatimonadetes*, *Actinobacteria*) occurred sporadically in low abundances (≤1.5%).

Within *Proteobacteria* in *S*. *aurata*, *Betaproteobacteria* was the dominant class in four locations (Yaltra, Chania, Chios, Igoumenitsa; Greece), while in Atalanti, *Betaproteobacteria* and *Gammaproteobacteria* co-dominated, with 22.1% and 23.7%, respectively (Figure S4). Other than this, *Gammaproteobacteria* was the second most abundant class of *Proteobacteria*, with *Alphaproteobacteria* always in low abundances (Figure S4). On the contrary, in *D*. *labrax*, *Gammaproteobacteria* dominated in three locations (Chania, Yaltra, Atalanti; Greece) followed by *Betaproteobacteria* and *Alphaproteobacteria*. In the rest of the locations (Igoumenitsa and Chios; Greece), *Betaproteobacteria* was the dominant taxon. In general, *Alphaproteobacteria* abundances in *D. labrax* were higher than in *S*. *aurata* (Figure S4). The most abundant orders in all locations for both host species were the *Micrococcales*, *Corynebacteriales*, *Propionibacteriales*, *Bifidobacteriales*, *Flavobacteriales*, *Bacteroidales*, *Bacillales*, *Lactobacillales*, *Burkholderiales* and *Pseudomonadales*.

**Figure 1:**
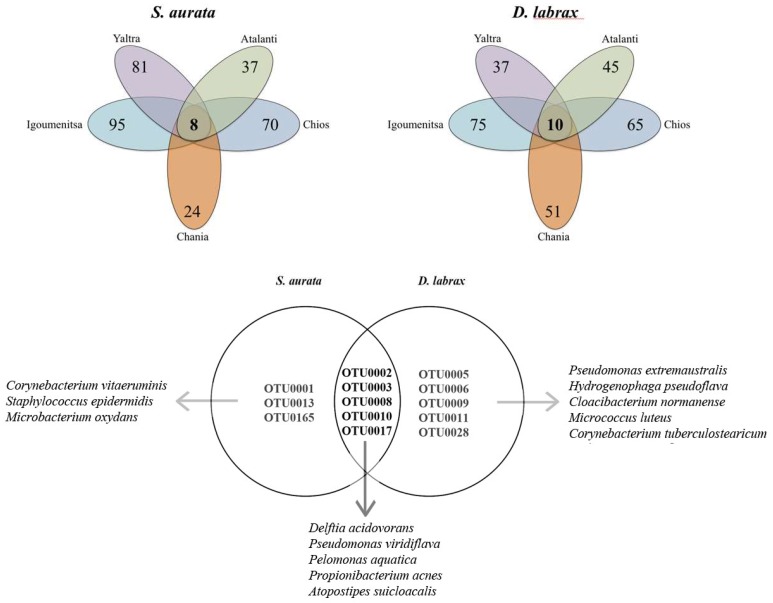
Flower diagram of the shared operational taxonomic units (OTU) between *Sparus aurata* and *Dicentrarchus labrax* individuals from different aquaculture sites in Greece.

A small set of OTUs was found to occur in all individuals from all five locations (8 in *S*. *aurata* and 10 in *D*. *labrax*), i.e., representing the core mid gut microbiota (sensu [3]) for each species (Figure 1). Moreover, five of these OTUs (Figure 1) were shared between the two species. The closest phylogenetic relatives for these OTUs were *Delftia acidovorans* (*Burkholderiales*), *Pseudomonas panacis* (*Pseudomonadales*), *Pelomonas puraquae* (*Burkholderiales*), *Propionibacterium acnes* (*Propionibacteriales*) and *Atopostipes suicloacalis* (*Lactobacillales*). (Table S3). The estimation of the shared OTUs doubling time (based on the 16S rDNA gene copy number [38] ranged between 0.8 and 2.0 h^-1^ (Table S3), implying that they represent bacteria which can grow fast in the fish GIT and thus, they are more likely to outcompete other bacterial taxa.

*Delftia* spp. have been previously retrieved from fish gut of healthy grouper [39], rainbow trout [40], 2012), and Atlantic salmon [41] individuals. The members of this genus are strictly aerobic and chemo-organotrophic but not fermentative [42]. *Pseudomonas* spp. have been isolated from several fish species and have been evaluated as potential probiotics in aquaculture industry [43,44,45]. *Pelomonas* sp. could be a resident GITM as it has been found in the gut of farmed fish [46]. *Propionibacterium acnes* is commonly found in fish [47,48,49,50,21] and snails [51] but its major importance for the human skin microbiome [52] renders it as an uncertain autochthonous gut symbiont for *S. aurata* and *D. labrax*. *Atopostipes* is a fermentative genus and to date it has been associated with fermented flesh of skate (*Raja pulchra*) [53] but also with the Atlantic salmon (*Salmo salar*) gut [49]. Thus, it is likely a bacterium with potential fermentative role in farmed *S. aurata* and *D. labrax*.

*S. aurata* shared OTUs belonged to taxa (*Burkholderiales, Pseudomonadales*, *Flavobacteriales, Actinobacteria*) reported in wild, organic and conventionally reared *S. aurata* individuals [48] while the identified closest relatives of these OTUs have been previously retrieved from similar environments (Table S3). This further suggests that these bacteria could be members of *S*. *aurata* core bacterial community.

The observed core bacterial community for both species consisted mostly from nonsporulating, mesophilic bacteria, with diverse types of respiration with some of them presenting important features in other animals. For example, *Micrococcus luteus* possesses anti-*Vibrio* activity in the freshwater fish *Oreochromis niloticus*; *Pseudomonas panacis* degrades cellulose in the gut of the bark beetles *Dendroctonus armandi,* while *P*. *veronii* has been reported to have metabolic pathways related to central carbohydrate metabolism, nutrients uptake and plant hormone auxin production in the grapevine, *Vitis vinifera*, root [54]. Most of the rest core gut bacterial OTUs, in *S*. *aurata* and *D. labrax* were assigned to similar orders such as *Corynebacteriales, Pseudomonadales* and *Micrococcales,* though in different species (Table S2). Most of them have been retrieved from similar isolation sources (e.g., stool, intestine, manure) (Table S3). The number of OTUs occurring only in one location varied between 11–61 and 22–55 for *S. aurata* and *D. labrax*, respectively (Figure S4).

Geographic distance between the aquaculture farms did not show any correlation with the gut bacterial community structure for both species (Figure S5) and nonmetric multidimensional scaling (NMDS, Figure S6) based on the Bray–Curtis distance of presence/absence OTUs, showed no clear geographic separation (ANOSIM using Euclidean distances *p* = 0.391, *R* = 9.3^−5^) of the gut bacterial communities for both species as well. This implies, that the observed GITM structure for each of the two fish species investigated in this study are not related to the vicinity of the aquaculture farms.

**Figure 2:**
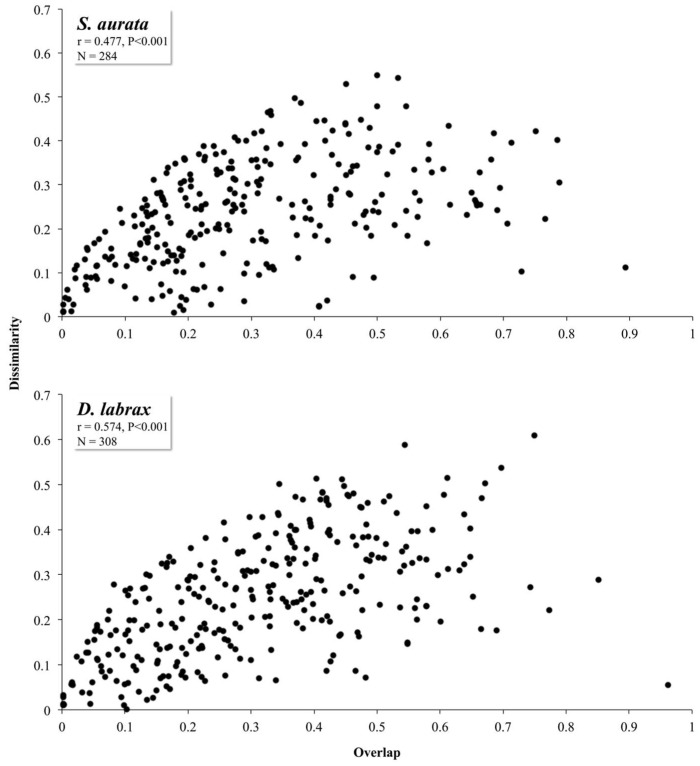
Dissimilarity vs. overlap correlation of all the possible sample pairs of the gut bacterial operational taxonomic units between different *Sparus aurata* and *Dicentrarchus labrax* aquaculture sites in Greece.

In the current study, the correlations between the overlap and dissimilarity of GITM communities structure were positive for both fish species considered (*r* = 0.477 and 0.574 for *p* < 0.002 in *S*. *aurata* and *D*. *labrax,* respectively) (Figure 2), suggesting high inter-individual variability in terms of OTUs abundances even in the same location. Similar results have also been observed in fecal microbiota for both *S*. *aurata* and *D*. *labrax* [55,56]. While in human gut microbiome, the inter-individual variability is more easily understood due to parameters such as dietary patterns and personal interests [57,58], here we concluded that inter-individual variability in the autochthonous gut bacteria of *D*. *labrax* and *S. aurata*, is more likely related with individual genetic factors. The observed inter-individual variability means that the gut microenvironment of these two host fish species promotes selective pressure in the bacterial communities. However, while the overlap of these bacterial communities increases, the same happens with dissimilarity, indicating host-independent parameters also shaping gut bacterial community in human [18,59] and fish GITM [60,61].

The most prominent factors promoting the inter-individual microbiota variation have only recently been taken into account and these are host genotype, gut colonization during the early developmental stages, environmental effects on GITM acquisition, diet, diseases and respective medication [8]. One reason for the GITM inter-individual variability is that caged fish are fed mechanically, a way that does not secure equal food consumption for each fish due to individual differences in their activity. The extent of GITM individual variability is important to know for the following reasons: (a) it dictates the number of replicate samples per species that need to be analyzed [58], (b) it helps distinguishing between autochthonous (resident) bacteria which colonize the gut mucosa and the allochthonous (transient) bacteria occurring mostly in the digesta [9,62]. The demonstrated individual host variability could be the reason for the low number of shared OTUs in both allopatric populations studied here, but larger datasets are required in order to fully unravel this issue.

**Figure 3:**
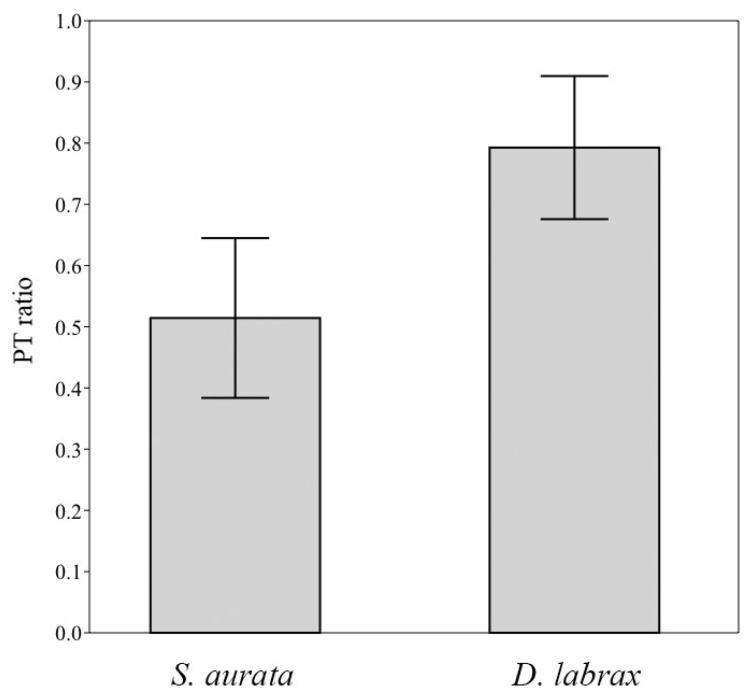
Ratio of positive to total (PT) correlations (Pearson, −0.7 > *R* > 0.7, *p* < 0.05) of the most dominant operational taxonomic units (OTUs) in *Sparus aurata* and *Dicentrarchus labrax* individuals from aquaculture sites in Greece. Vertical lines indicate standard error.

Although one-way analysis of variance (ANOVA) revealed no statistically significant differences between gut bacterial communities for both species (*p* > 0.05), (Figure S3), the biological relations of the bacterial communities were different. The ratio of positive to total (PT) correlations of the most dominant OTUs of *S*. *aurata* and *D*. *labrax* individuals was significantly different (*p* < 0.05), suggesting different biological relationships in the guts of the two species (Figure 3). The high ratio of the positive to total (PT) correlations of the most dominant operational taxonomic units demonstrates that the majority of the dominant bacteria have either cooperative interactions or, at least, they do not participate in competitive nutrition. Such relationships in microbial populations are believed to be beneficial to the host as they ensure high capacity of utilizing the complex array of available substrates found in the gut [63,35,64].

## 4. Conclusions

It is still unknown whether and how the gut microbial communities of fish can contribute nutrients and energy to the host and maintain a balance with the fish’s metabolism and immune system. This study presents evidence for core gut bacterial communities within the two examined host species (*S. aurata* and *D. labrax*), and also a small set of OTUs that have been found in common between them, indicating that some autochthonous gut bacterial representatives of the *Delftia*, *Pseudomonas*, *Pelomonas*, *Propionibacterium* and *Atopostipes* genera can colonize different host species. Despite the inter-individual variability and the distance of each farm location, there is no significant difference between the gut bacterial communities in the two host species. The results also revealed these gut bacterial communities form different biological relations between their members as revealed by their populations association networks.

## Figures and Tables

**Table 1:** Pyrosequencing results of the bacterial 16S rDNA gene diversity in the midgut of *Sparus aurata* and *Dicentrarchus labrax* individuals (N) from different aquaculture sites in Greece. OTUs: operational taxonomic units; N: number of individual midgut samples analyzed.

Site	Reads		OTUs		No. of the Most Dominant OTUs (Cumulative Relative Dominance ≥ 80%)		Most Abundant OTU, Dominance (%) and Closest Relative (≥97%)	
	*S. aurata*	*D. labrax*	*S. aurata*	*D. labrax*	*S. aurata*	*D. labrax*	*S. aurata*	*D. labrax*
Chania	827 ± 512.4 *N* = 4	2395 ± 725.4 *N* = 5	11 ± 2.2	16 ± 8.7	10 (80.0)	17 (79.9)	OTU0011 (22.7) *Micrococcus luteus*	OTU0014 (17.2) *Paracocccus denitrificans*
Igoumenitsa	2360 ± 1972.7 *N* = 5	1809 ± 571.3 *N* = 4	25 ± 28.3	27 ± 20.3	13 (81.0)	14 (80.0)	*OTU0004* (27.8) *Bacillus hisashii*	OTU0001 (19.9) *Corynebacterium vitaeruminis*
Chios	2407 ± 1771.0 *N* = 6	2148 ± 1785.2 *N* = 6	18 ± 11.4	17 ± 9.8	13 (79.5)	13 (80.9)	OTU0004 (22.0) *Bacillus hisashii*	OTU0001 (24.7) *Corynebacterium vitaeruminis*
Yaltra	2656 ± 1529.0 *N* = 6	697 ± 367.3 *N* = 6	19 ± 12.2	11 ± 2.4	21 (80.0)	10 (79.9)	OTU0002 (16.9) *Delftia acidovorans*	OTU0025 (21.9) *Acinetobacter lwoffii*
Atalanti	1574 ± 1005.9 *N* = 4	2533 ± 1052.7 *N* = 5	13 ± 6.1	14 ± 8.1	12 (80.1)	11 (80.0)	OTU0005 (14.7) *Pseudomonas extremaustralis*	OTU0002 (17.7) *Delftia acidovorans*

## Supplementary Materials

The following are available online at http://www.mdpi.com/2076-2607/6/3/92/s1, Table S1. Body weight of the *Sparus aurata* and *Dicentrarchus labrax* individuals used in this study; Table S2. Ingredients of the diets used at the time of sampling; Table S3. Bacterial 16S rDNA operational taxonomic units (OTU) found in the midgut of commercially reared *Sparus aurata* and *Dicentrarchus labrax* individuals from different aquaculture sites in Greece; Figure S1. Aquaculture sampling sites. I: Igoumenitsa, Y, Yaltra, A: Atalanti, Ch: Chios, C: Chania; Figure S2. Rarefaction curves bacterial operational taxonomic units generated by16S rDNA tag pyrosequencing from the midgut of *Sparus aurata* and *Dicentrarchus labrax* individuals originating from different aquaculture farms in Greece; Figure S3. Box-plot of the bacterial operational taxonomic units found in the midgut of *Sparus aurata* and *Dicentrarchus labrax* individuals originating from different aquaculture farms in Greece; Figure S4. Taxonomy (phyla: top row; *Proteobacteria* sub-phyla: bottom row) of the found bacterial operational taxonomic units found in the midgut of *Sparus aurata* and *Dicentrarchus labrax* individuals originating from different aquaculture farms in Greece; Figure S5. Relationship of the shared operational taxonomic units (OTUs) and the total number of OTUs with the distance between different *Sparus aurata* and *Dicentrarchus labrax* aquaculture sites in Greece; Figure S6. Non-metric multidimensional scaling (NMDS) based on the gut bacterial operational taxonomic units between *Sparus aurata* and *Dicentrarchus labrax* individuals from different aquaculture sites in Greece. Red and blue lines include all *S. aurata* and *D. labrax* samples, respectively.
