# Supplementary material for: Gut Bacterial Communities in Geographically Distant Populations of Farmed Sea Bream (Sparus aurata) and Sea Bass (Dicentrarchus labrax)
Source: Microorganisms. 2018 Sep 1;6(3):92. doi: 10.3390/microorganisms6030092 (PMC6164763; doi:10.3390/microorganisms6030092)
Supplement: Supplementary file 1 [file microorganisms-06-00092-s001.pdf]

Gut bacterial communities in geographically distant populations of farmed sea bream (*Sparus aurata*) and sea bass (*Dicentrarchus labrax*)

Eleni Nikouli[1], Alexandra Meziti[1], Efthimia Antonopoulou[2], Eleni Mente[1], Konstantinos Ar. Kormas[1*]

[1] Department of Ichthyology and Aquatic Environment, School of Agricultural Sciences, University of Thessaly, 384 46 Volos, Greece

[2] Laboratory of Animal Physiology, Department of Zoology, School of Biology, Aristotle University of Thessaloniki, 541 24 Thessaloniki, Greece

* Corresponding author; Tel.: +30-242-109-3082, Fax: +30-242109-3157, E-mail: kkormas@uth.gr, kkormas@gmail.com

Supplementary material

Table S1. Body weight of the *Sparus aurata* and *Dicentrarchus labrax* individuals used in this study.

		Chania		Chios		Igoumenitsa		Yaltra		Atalanti	
**Sample**	**Body weight (g)**	*S. aurata*	*D. labrax*	*S. aurata*	*D. labrax*	*S. aurata*	*D. labrax*	*S. aurata*	*D. labrax*	*S. aurata*	*D. labrax*
1		359	378	558	420	433	448	481	346	260	785
2		355	294	579	442	493	556	516	397	240	340
3		376	275	468	554	450	464	540	415	440	500
4		392	395	530	460	440	483	492	493	365	860
5		420	362	483	479	542		492	406		995
6				521	505			506	461		
	Mean	380.40	340.80	523.17	476.67	471.60	487.75	504.50	419.67	326.25	696.00
	SEs	11.89	23.76	17.36	19.56	20.46	23.85	8.68	21.00	46.79	120.29

Table S2. Ingredients of the diets used at the time of sampling.

**Ingredient**	***Sparus aurata*** **(6 mm; 350-450 g)****	***Dicentrarchus labrax*** **(6 mm; 450-800 g)****
Crude proteins (%)	42 – 44	37 – 39
Crude lipids (%)	19 – 21	20 – 22
Nitrogen free extract (NFE) (%)	20 – 26	19 – 25
Crude cellulose (%)	1 – 3	2 – 4
Ash (%)	5.8 – 7.8	6.2 – 8.2
Total P (%)	0.7 – 0.9	0.8 – 1.0
Gross energy (MJ/Kg)	21.5 – 23.5	20.6 – 22.6
Classical digestible energy* (MJ/Kg)	19.5	18.9
Added vitamin D3 (I.U./Kg)	500	500
Added vitamin E (I.U./Kg)	180	100
Added vitamin C (I.U./Kg)	250	100
Feeding rate (%), i.e. Kg of fish feed/100 Kg of fish biomass per day	0.7	0.7

* Classical digestible energy calculated on proteins, lipids and NFE.
** Mean size of feed pellet; fish weight.

Table S3. Bacterial 16S rDNA operational taxonomic units (OTU) found in the midgut of commercially reared *Sparus aurata* and *Dicentrarchus labrax* individuals from different aquaculture sites in Greece.

: shared between *S. aurata* and *D. labrax* in all sites (core microbiota for *S. aurata* and *D. labrax*)
: shared between *S. aurata* individuals in all sites (core microbiota)
: shared between *D. labrax* individuals in all sites (core microbiota)
: most abundant in *S. aurata*
: most abundant in *D. labrax*

OTU	Closest relative	Similarity (%)	GenBank accession No.	Habitat of origin	Reference	Average ± SD number of 16S rRNA gene copies*
0002	*Delftia acidovorans* (Burkholderiales)	100	KX184216	*Triatoma dimidiata*	Unpublished	(N=1) x = 5
0003	*Pseudomonas panacis* (Pseudomonadales)	100	KF501476	*Dendroctonus armandi* gut	Unpublished	*Pseudomonas* spp. (N=247) x = 4.8 ± 1.3
0008	*Pelomonas puraquae* (Burkholderiales)	100	JQ659646	plant tissue	[65]	(N=) x = ±
0010	*Propionibacterium acnes* (Propionibacteriales)	100	KF933807	Homo sapiens oral cavity	Unpublished	(N=11) x = 2.9 ± 0.3
0017	*Atopostipes suicloacalis* (Lactobacillales)	97.1	NR_028835	Underground swine manure storage pit	[66]	(N=1) x = 2
0001	*Corynebacterium spheniscorum* (Corynebacteriales)	98.9	NR_027201	wild penguins cloacae	[67]	*Corynebacterium* spp. (N=152) x = 4.4 ± 0.8

0013	*Staphylococcus epidermidis* (Bacillales)	100	KU550237	*Aedes albopictus* adult female midgut	Unpublished	(N=7) $x = 5.7 \pm 0.5$
0165	*Microbacterium phyllosphaerae* (Actinomycetales)	99.1	LT223598	Human stool	Unpublished	*Microbacterium* spp. (N=11) $x = 2 \pm 0.4$
0005	*Pseudomonas veronii* (Pseudomonadales)	100	KJ726603	channel catfish intetsine	Unpublished	(N=1) $x = 6$
0006	*Hydrogenophaga atypica* (Burkholderiales)	98.4	KT345668	hot spring	Unpublished	Hiydrogenophaga spp. (N=3) $x = 1.7 \pm 0.5$
0009	*Cloacibacterium normanense* (Flavobacteriales)	100	NR_042187	municipal wastewater	[68]	(N=1) $x = 4$
0011	*Micrococcus luteus* (Micrococcales)	100	LN998081	Mammoth stool specimen	Unpublished	(N=1) $x = 2$
0028	*Corynebacterium tuberculostearicum* (Corynebacteriales)	100	LN867524	Urine of Patient with kidney stone	[69]	*Corynebacterium* spp. (N=152) $x = 4.4 \pm 0.8$
0004	*Bacillus thermoamylovorans* (Bacillales)	99.7	KR364739	Mouse gut	[70]	(N=4) $x = 2.5 \pm 1.3$
0007	*Enterobacter cloacae* (Enterobacterales)	100	KX674047	Human fecal sample	Unpublished	(N=27) $x = 8 \pm 0.2$
0012	Clone HAW-RM37-2-B-1209d-A8	100	FN563272	Mesophilic biogas digester	[71]	

0014	*Paracoccus chinensis* (Rhodobacterales)	100	KT899804	Heterotrophic bacterium from Arctic region	Unpublished	(N=5) $x = 3 \pm 0.6$
0015	*Stenotrophomonas maltophilia* (Xanthomonadales)	100	AB167179	chemostat enrichment at day3, constructed with an aquifer soil as inoculum and phenol was supplied as sole carbon source	[72]	(N=11) $x = 4 \pm 0$
0016	*Bifidobacterium thermophilum* (Bifidobacteriales)	100	HQ851038	Calves digestive tract	[73]	(N=1) $x = 4$
0020	*Ralstonia insidiosa* (Burkholderiales)	100	KF378751	oil-contaminated soil	Unpublished	(N=2) $x = 3$
0022	Clone SGR163	98.7	JQ793519	rhizospheric soil	[74]	
0023	Clone nck113g05c1	100	KF089262	Homo sapiens skin, antecubital fossa	[75]	
0024	*Sphaerotilus natans* (Burkholderiales)	100	GU591793	ferrous iron-containing cold mountainous spring	Unpublished	(N=1) $x = 1$
0025	*Acinetobacter lwoffii* (Pseudomonadales)	100	LT223613	Human stool	Unpublished	(N=1) $x = 6$
0026	*Petrobacter succinimandens* (Hydrogenophilales)	100	AY219714	oil well	Unpublished	
0027	*Pseudomonas pseudoalcaligenes*	100	KF436943	*Mesodesma donacium* gut	[76]	(N=3) $x = 4.3 \pm 0.5$

0028	*Corynebacterium tuberculostearicum* (Corynebacteriales)	100	LN867524	Urine of patient with kidney stone	[69]	*Corynebacterium* spp. (N=169) x = 4.4 ± 0.8
0030	*Comamonas testosteroni* (Burkholderiales)	100	KM277791	Active sludge obtained from a wastewater treatment plant of pertrochemical company	Unpublished	(N=3) x = 4.7 ± 3.1
0031	*Bacillus tequilensis* (Bacillales)	100	KC992298	Crude oil contaminated soil	Unpublished	(N=2) x = 2
0032	*Salinicoccus roseus* (Bacillales)	100	AF237976	Cultured marine eubacterium; marine snow isolate from >20 m depth from Northern Adriatic Sea	[77]	
0033	*Pseudomonas alkylphenolia* (Pseudomonadales)	100	KU570339	*Eriocheir sinensis* intestinal tract	Unpublished	
0037	*Bacillus* sp. DVH100	99.7	KX785123		Unpublished	*Bacillus* spp. (N=370) x = 10.5 ± 2.4
0041	*Sphingomonas kyeonggiensis* (Sphingomonadales)	100	NR_134182	Ginseng field soil	[78]	
0042	*Bacillus beringensis*	100	KJ575017	Deep sea sediment	Unpublished	
0043	*Bacillus* sp. 9-3AIA (Bacillales)	94.8	FN397519	*Homo sapiens* faeces	[79]	*Bacillus* spp. (N=370) x = 10.5 ± 2.4
0044	*Comamonas aquatica* (Burkholderiales)	100	FJ493173	Polluted farmland	Unpublished	(N=3) x = 1.7 ± 1.1

0045	*Paenibacillus phoenicis* (Bacillales)	99.7	NR_108292	Kennedy Space Center clean-room floor	[80]	
0046	*Pedomicrobium ferrugineum* (Rhizobiales)	100	NR_104840		Unpublished	
0047	*Pseudomonas luteola* (Pseudomonadales)	100	KM891563	*Delphinium tenii* floral nectar	Unpublished	
0049	*Agrobacterium tumefaciens* (Rhizobiales)	100	KU159273	Plant nodule	Unpublished	(N=4) $x = 4 \pm 0.7$
0051	*Daeguia caeni* (Rhizobiales)	100	NR_044269	Sludge of a textile dyeworks	[81]	

* From Microbial Genome Resources (https://www.ncbi.nlm.nih.gov/genomes/MICROBES/microbial_taxtree.html) or *rrn*DB (https://rrndb.umms.med.umich.edu/), accessed, 01/04/2018.

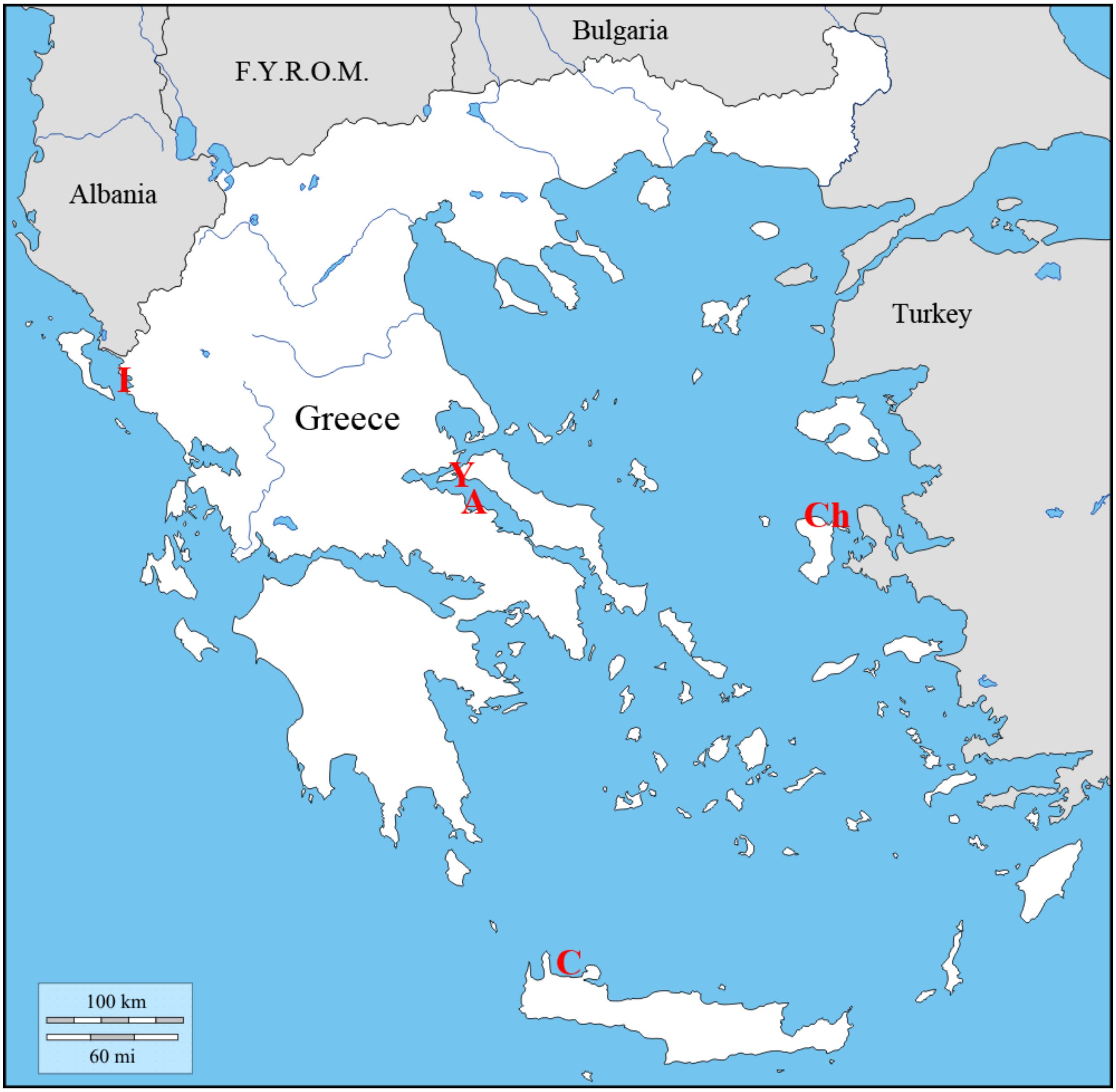

Figure S1. Aquaculture sampling sites. I: Igoumenista, Y, Yaltra, A: Atalanti, Ch: Chios, C: Chania.

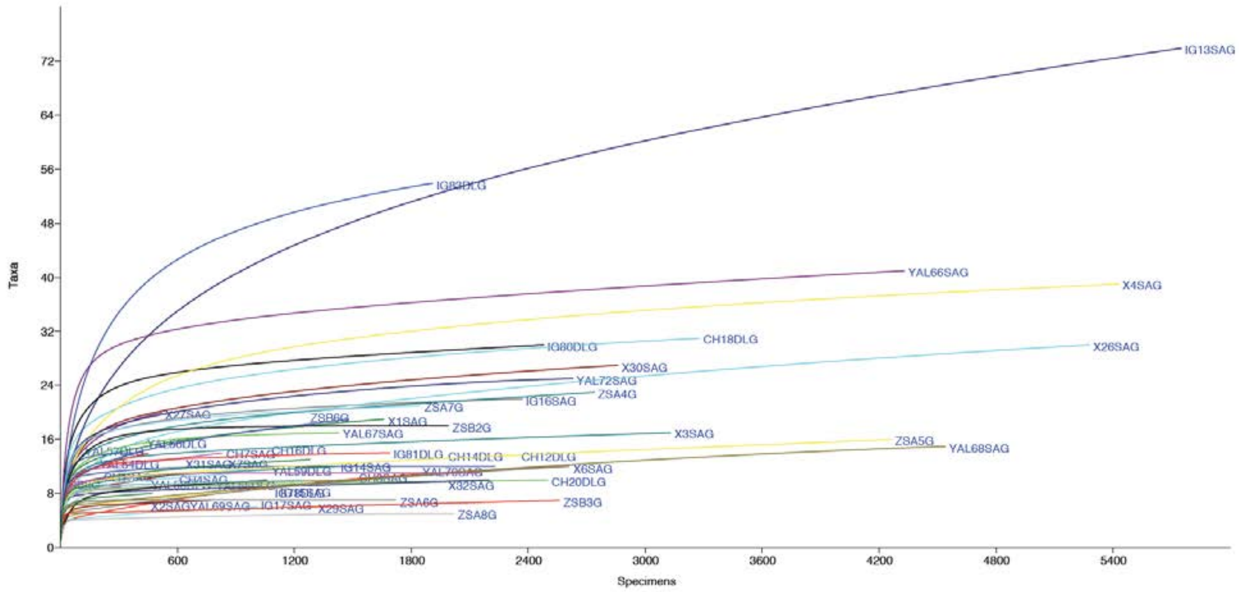

Figure S2. Rarefaction curves bacterial operational taxonomic units generated by16S rDNA tag pyrosequencing from the midgut of *Sparus aurata* and *Dicentrarchus labrax* individuals originating from different aquaculture farms in Greece.

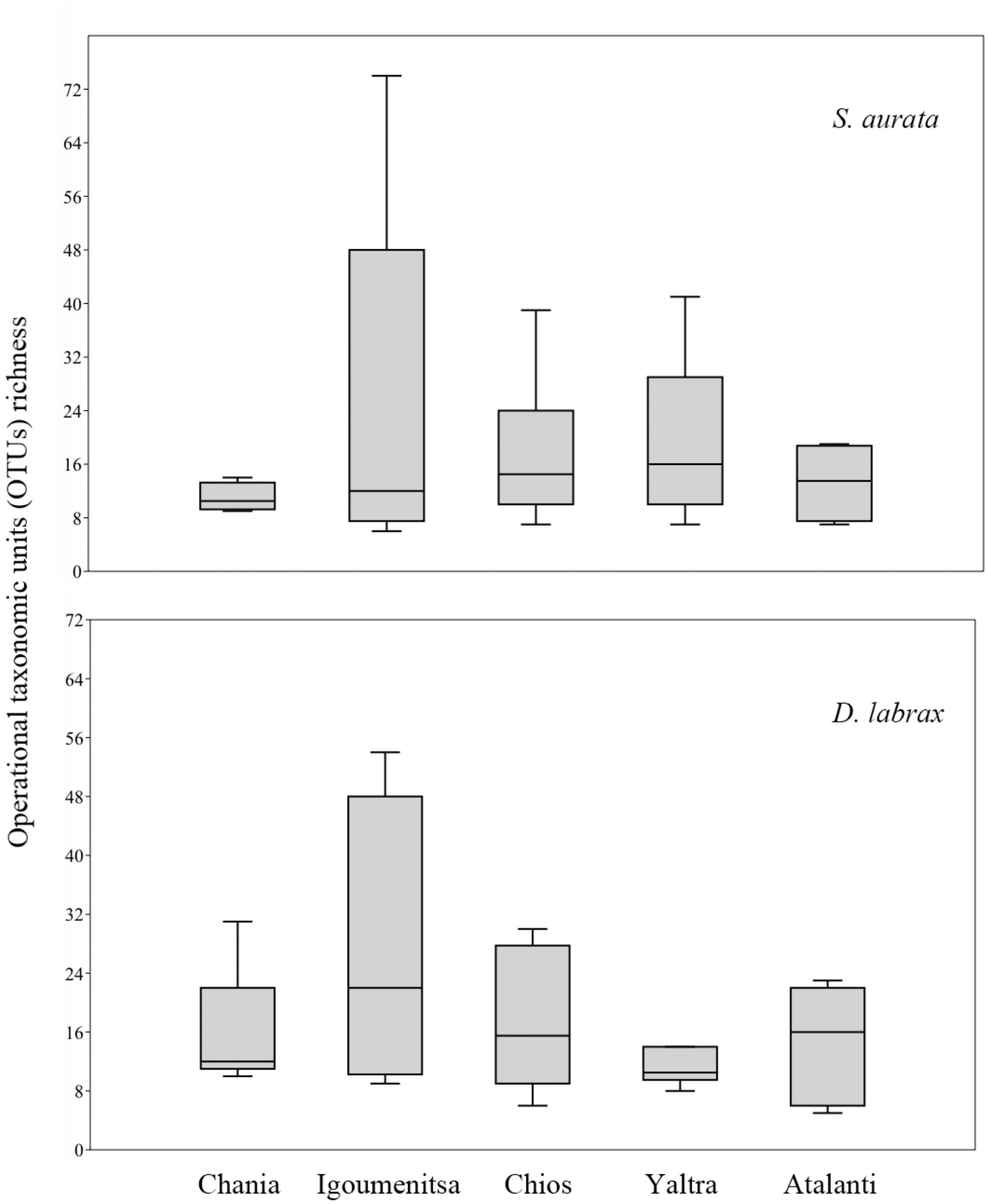

Figure S3. Box-plot of the bacterial operational taxonomic units found in the midgut of *Sparus aurata* and *Dicentrarchus labrax* individuals originating from different aquaculture farms in Greece.

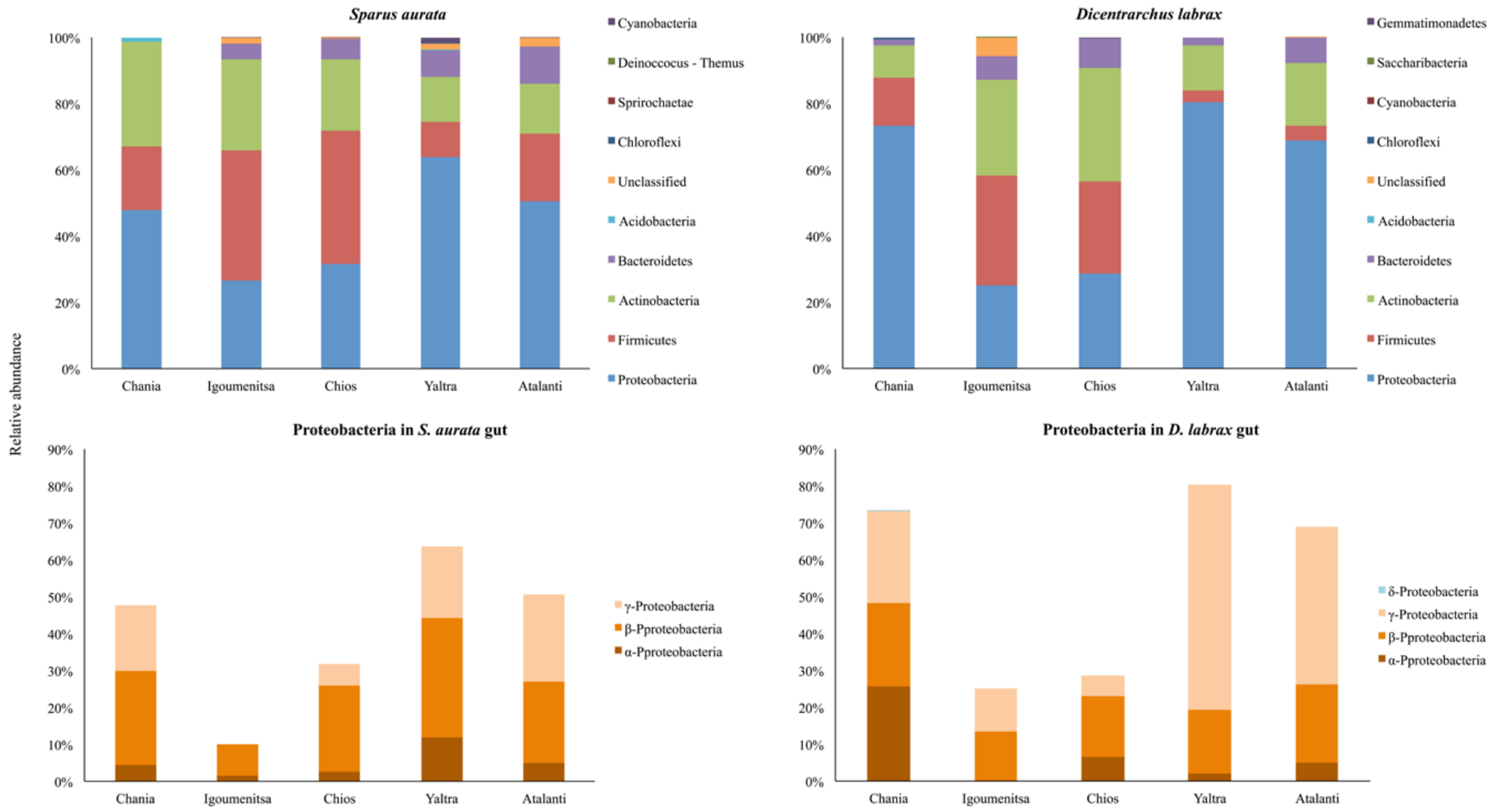

Figure S4. Taxonomy (phyla: top row; Proteobateria sub-phyla: bottom row) of the found bacterial operational taxonomic units found in the midgut of *Sparus aurata* and *Dicentrarchus labrax* individuals originating from different aquaculture farms in Greece.

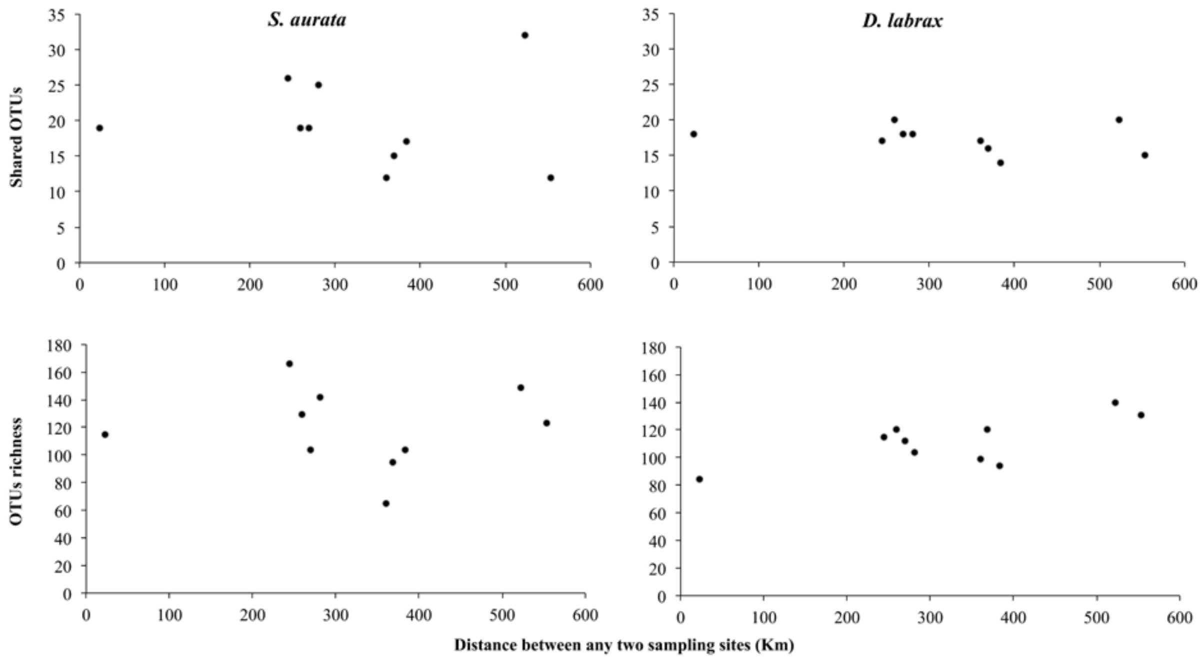

Figure S5. Relationship of the shared operational taxonomic units (OTUs) and the total number of OTUs with the distance between different *Sparus aurata* and *Dicentrarchus labrax* aquaculture sites in Greece.

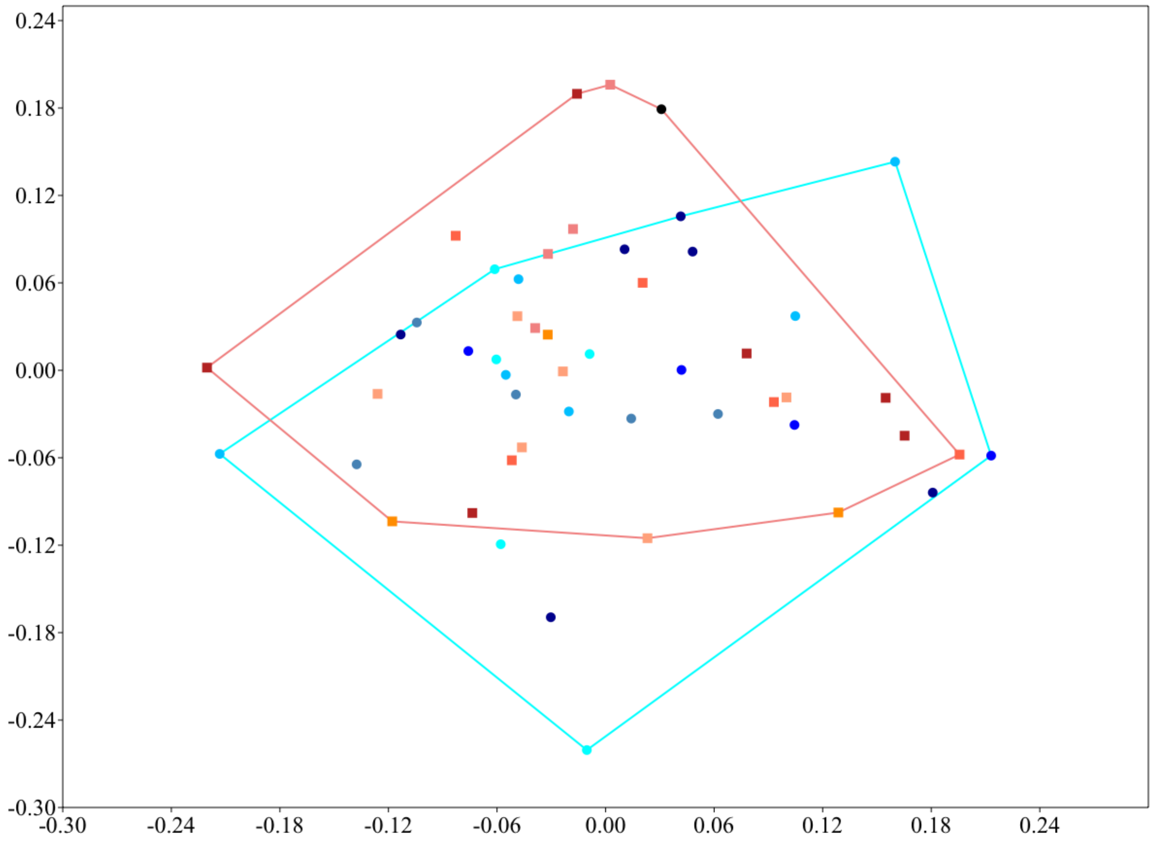

Figure S6. Non-metric multidimensiosal scaling (NMDS) based on the gut bacterial operational taxonomic units between *Sparus aurata* and *Dicentrarchus labrax* individuals from different aquaculture sites in Greece. Red and blue lines include all S. aurata and D. labrax samples, respectively.

References

65. Madhaiyan, M.; Alex, T.H.; Ngoh, S.T.; Prithiviraj, B.; Ji, L. Leaf-residing methylobacterium species fix nitrogen and promote biomass and seed production in *Jatropha curcas*. *Biotechnol. Biofuels* **2015**, *8*, 222. doi: 10.1186/s13068-015-0404-y.
66. Cotta, M.A.; Whitehead, T.R.; Collins, M.D.; Lawson, P.A. *Atopostipes suicloacale* gen. nov., sp. nov., isolated from an underground swine manure storage pit. *Anaerobe* **2004**, *10*, 191–195. doi: 10.1016/j.anaerobe.2004.04.001.
67. Goyache, J.; Vela, A.I.; Collins, M.D.; Ballesteros, C.; Briones, V.; Moreno, J.; Yorio, P.; Domínguez, L.; Hutson, R.; Fernández-Garayzábal, J.F. *Corynebacterium spheniscorum* sp. nov., isolated from the cloacae of wild penguins. *Int. J. Syst. Evol. Microbiol*. **2003**, *53*, 43–46. doi: 10.1099/ijs.0.02343-0.
68. Allen, T.D.; Lawson, P.A.; Collins, M.D.; Falsen, E.; Tanner, R.S. *Cloacibacterium normanense* gen. nov., sp. nov., a novel bacterium in the family *Flavobacteriaceae* isolated from municipal wastewater. *Int. J. Syst. Evol. Microbiol.* **2006**, *56*, 1311–1316. doi: 10.1099/ijs.0.64218-0.
69. Abd Al-Abbas, M.J.; Jasim, N.A. Isolation and identification of bacteria from urine of patient with kidney stone. *American Sci. Res. J. for Eng. Technol. Sci.* **2016**, *26*: 230-249.

70. Lagkouvardos, I.; Pukall, R.; Abt, B.; Foesel, B.U.; Meier-Kolthoff, J.P.; Kumar, N.; Bresciani, A.; Martinez, I.; Just, S.; Ziegler, C., et al. The mouse intestinal bacterial collection (miBC) provides host-specific insight into cultured diversity and functional potential of the gut microbiota. *Nat. Microbiol.* **2016**, *1*, 16131. doi: 10.1038/nmicrobiol.2016.131.
71. Krakat, N.; Schmidt, S.; Scherer, P. Potential impact of process parameters upon the bacterial diversity in the mesophilic anaerobic digestion of beet silage. *Bioresour. Technol*. **2011**, *102*, 5692–5701. doi: 10.1016/j.biortech.2011.02.108.
72. Futamata, H.; Nagano, Y.; Watanabe, K.; Hiraishi, A. Unique kinetic properties of phenol-degrading *Variovorax* strains responsible for efficient trichloroethylene degradation in a chemostat enrichment culture. *Appl. Environ. Microbiol.* **2005**, *71*, 904–911. doi: 10.1128/aem.71.2.904-911.2005.
73. Bunešová, V.; Vlková, E.; Rada, V.; Ročková, Š.; Svobodová, I.; Jebavý, L.; Kmeť, V. *Bifidobacterium* animalis subsp. lactis strains isolated from dog faeces. *Vet. Microbiol.* **2012**, *160*, 501–505. doi: 10.1016/j.vetmic.2012.06.005.
74. Yousuf, B.; Keshri, J.; Mishra, A.; Jha, B. Application of targeted metagenomics to explore abundance and diversity of CO_2-fixing bacterial community using *cbbL* gene from the rhizosphere of Arachis hypogaea. Gene **2012**, *506*, 18–24. doi: 10.1016/j.gene.2012.06.083.
75. Oh, J.; Freeman, A.F.; Park, M.; Sokolic, R.; Candotti, F.; Holland, S.M.; Segre, J.A.; Kong, H.H. The altered landscape of the human skin microbiome in patients with primary immunodeficiencies. *Genome Res*. **2013**, *23*, 2103–2114. doi: 10.1101/gr.159467.113.
76. Muñoz, C.; Hidalgo, C.; Zapata, M.; Jeison, D.; Riquelme, C.; Rivas, M. Use of cellulolytic marine bacteria for enzymatic pretreatment in microalgal biogas production. *Appl. Environ. Microbiol*. **2014**, *80*, 4199–4206. doi: 10.1128/aem.00827-14.
77. Moeseneder, M.M.; Winter, C.; Herndl, G.J. Horizontal and vertical complexity of attached and free-living bacteria of the Eastern Mediterranean Sea, determined by 16S rDNA and 16S rRNA fingerprints. *Limnol. Oceanogr.* **2001**, *46*, 95–107. doi: 10.4319/lo.2001.46.1.0095.
78. Son, H.-M.; Kook, M.; Tran, H.T.H.; Kim, K.-Y.; Park, S.-Y.; Kim, J.-H.; Yi, T.-H. *Sphingomonas kyeonggiense* sp. nov., isolated from soil of a ginseng field. *Antonie van Leeuwenhoek* **2014**, *105*, 791–797. doi: 10.1007/s10482-014-0122-7.
79. Hoyles, L., Honda, H., Logan N.A., Halket, G., La Ragione R.M., McCartney A.L. Recognition of greater diversity of *Bacillus* species and related bacteria in human faeces. *Res. Microbiol*. **2012**, *163*, 3–13. doi: 10.1016/j.resmic.2011.10.004.
80. Ghosh, S.; Osman, S.; Vaishampayan, P.; Venkateswaran, K. Recurrent isolation of extremotolerant bacteria from the clean room where phoenix spacecraft components were assembled. *Astrobiology* **2010**, *10*, 325–335. doi:10.1089/ast.2009.0396.
81. Yoon, J.H.; Kang, S.J.; Park, S.; Oh, T.K. *Daeguia caeni* gen. nov., sp. nov., isolated from sludge of a textile dye works. *Int. J. Syst. Evol. Microbiol*. **2008**, *58*, 168–172. doi: 10.1099/ijs.0.65483-0.
